# Recognizing the importance of COVID-19 data wrangling

**DOI:** 10.1172/JCI164375

**Published:** 2022-09-28

**Authors:** Laura J. Rasmussen-Torvik

The 2022 Lasker~Bloomberg Public Service award honors Dr. Lauren Gardner, who leads the team that built the Johns Hopkins COVID-19 global tracking map (https://coronavirus.jhu.edu/map.html). Established in January 2020, the map evolved into the Johns Hopkins Coronavirus Resource Center by March 2020. The map and resource center website, which are supported by Bloomberg Philanthropies and the Stavros Niarchos Foundation, were critical resources for scientists, national and international policymakers, the press, and the public in the early months of the COVID pandemic, and they remain important and widely used resources to this day. In addition to providing comprehensive, reliable, and easily digestible data at a key time in the COVID pandemic, Dr. Gardner’s work provides a model of how researchers and public health agencies should collect, process, and disseminate data during future infectious disease outbreaks.

## Descriptive epidemiology resources prior to COVID-19

Descriptive epidemiology is fundamental and describes the distribution of disease across persons, including sociodemographic categories, places, and time. Although these analyses don’t use complex statistics (often relying simply on counts or percentages), they do involve tremendous effort to acquire and clean the data and to harmonize data across different sources. Due to this volume of labor, and perhaps to the perceived lesser prestige of descriptive analyses relative to analytic epidemiology analyses (which seek to identify risk factors that contribute to our understanding of disease etiology), descriptive epidemiology research is performed primarily by public health agencies and advocacy groups. Prior to the COVID-19 pandemic, there were no well-publicized resources rapidly updating information about infectious diseases for the public (or scientific community). The CDC’s National Respiratory and Enteric Virus Surveillance System (https://www.cdc.gov/surveillance/nrevss/index.html) and FluView (https://www.cdc.gov/flu/weekly/index.htm) still update only weekly, with frequent revisions after initial postings, and the World Health Organization FluNet (https://www.who.int/tools/flunet/flunet-summary) provides updates only biweekly. The Biosense platform utilized by the National Syndromic Surveillance Program (https://www.cdc.gov/nssp/overview.html#bioSense) is not publicly accessible. These systems were not prepared to deliver the frequent updates needed during the COVID-19 pandemic.

## The COVID website is born

In mid-January of 2022, Ensheng “Frank” Dong, a first-year PhD student in Civil and Systems Engineering working with Dr. Gardner at the Center for Systems Science and Engineering, was growing increasingly concerned about first-hand reports from his family concerning the emerging epidemic in China (1). At this point, the disease did not even have an official name, but after discussing the issue with Gardner, Dong worked over the course of an afternoon and evening to create the first version of the COVID-19 map, repurposing some of his existing work with Gardner on predicting measles hot spots in the US and utilizing ArcGIS mapping technology (1, 2). The website went live on January 22, 2022, with the intended audience of the research community — epidemiologists and disease modelers (3). When they shared news of the dashboard on social media, word spread rapidly in the research, press, and lay communities, and, by that afternoon, the dashboard had been featured on numerous major news sites. As case counts grew and awareness increased nationally and internationally, traffic to the website continued to surge such that in March 2020 the page attracted more total visits than the websites of the CDC or the New York Times (1). During this time, it also served as a central data source for the US Department of Health and Human Services.

## Maintaining a disease dashboard in a rapidly growing pandemic

While setting up the initial COVID-19 map may have been a relatively simple task for Dong and Gardner, maintaining and updating the resource during a rapidly expanding pandemic turned into a herculean effort. The dashboard initially used data from a single Chinese website (https://portal.dxy.cn/), but as the pandemic exploded, the team working on the dashboard expanded in order to collate and validate data from a vast and growing number of worldwide sources. These sources extended to social media, Facebook and Twitter, and news and media announcements in addition to data from international, national, and local health agencies (4). By July 2020, the team was collecting case reports from more than 3,500 locations, while updating the dashboard hourly. Collaborators at Johns Hopkins Applied Physics Laboratory created code for the team that could scrape machine-readable data from websites (5), but nonetheless required manual input to validate data sourcing and collection methods. As of March 2022, some of the data the team aggregated was still not machine readable (4). To this day, the website maintains a list of constantly updated data notes (https://coronavirus.jhu.edu/region-data-notes) to help people understand variations in data collection methods that may impact presented case counts around the world. Since February 2020 (5), Gardner’s team has also published all data underlying the dashboard in a GitHub repository (6), which, in addition, to making the underlying updated data available to the scientific community, also maintains a file history.

## Moving beyond the dashboard

Dr. Gardner’s research expertise extends far beyond the basic measurement of disease over persons, places, and time. She is at the forefront of global epidemiological risk assessment, integrating mathematical modeling and network optimization to explore the diverse factors that contribute to virus diffusion. Gardner has utilized this expertise to work on many modeling projects since the start of the pandemic. She has, for example, participated in work examining the early COVID-19 epidemic in Louisiana that uncovered the impact of large-scale superspreading events during the early outbreak in the US (7) and research comparing the transmissibility of SARS-CoV-2 variants Alpha and Iota (8). She’s also written about the limitations of cell phone data to model COVID-19 transmission in the US (9). Based on her experiences creating the COVID-19 dashboard, Gardner has advocated for the use of open public data standards and criteria for how data on infectious diseases are collected, reported, and shared (10). She has emphasized the need for immediate access to data, in machine-readable formats, that can be used by public health experts for planning and modeling as well as by the general public at large (10). She is also participating in efforts to examine the quality of prospective COVID-19 modeling during the pandemic and has recently released a preprint systematically reviewing over 100 papers with data-driven modeling studies on population-level dynamics of COVID-19 (11).

## The legacy of the COVID-19 dashboard

Dr. Gardner has been recognized in the lay press for the key role the Johns Hopkins COVID-19 global tracking map played in providing critical information to the public, press, and to international, federal, and local policymakers in the early months of the COVID-19 pandemic. However, it is important not to ignore the vital contributions of her work to the scientific community as well. As of early August 2022, Google Scholar lists over 8,000 citations to the February 2020 paper introducing the dashboard in *The Lancet Infectious Diseases* (12). The first COVID-NET article (13) cites the Johns Hopkins COVID-19 global tracking map for the estimate of current COVID-19 cases worldwide.

Perhaps the greatest demonstration of the importance and impact of Gardner’s work can be seen in the CDC response to the multicountry monkeypox outbreak in nonendemic countries identified by the WHO in May 2022 (https://www.who.int/emergencies/disease-outbreak-news/item/2022-DON385). As of mid-July 2022, the CDC had both a US and global case count map that were very similar in form and function to the COVID-19 global tracking map (https://www.cdc.gov/poxvirus/monkeypox/response/2022/index.html), updated daily (Monday–Friday). The COVID-19 dashboard not only improved our ability to respond to the community spread of SARS-COV-2 infections, but marks a fundamental turning point in how to approach infectious disease outbreaks.

The 2022 Lasker~Bloomberg Public Service Award recognition of Dr. Gardner spotlights the importance of data wrangling and the value of timely, accurate data. It is hard to envision navigating the last 2.5 years without the broad reach of the Johns Hopkins global tracking map, and we salute Dr. Gardner and her forward thinking in establishing and maintaining this unparalleled resource.

## Version 1 09/28/2022

Electronic publication

## Version 2 10/03/2022

Electronic publication
